# Torsades de Pointes in Severe Aortic Stenosis: A Case Report of Nightmare Double-trouble

**DOI:** 10.19102/icrm.2026.17102

**Published:** 2026-10-15

**Authors:** Pramod Kumar, Nikhat Parween, Sudipta Mondal, Nadeem Afroz Muslim

**Affiliations:** 1 Department of Cardiology, Sree Chitra Tirunal Institute for Medical Sciences and Technology, Thiruvananthapuram, India; 2 Neotia Getwel Multispecialty Hospital, Siliguri, India; 3 Department of Cardiology, The Mission Hospital, Durgapur, India

**Keywords:** Bradycardia-dependent arrhythmia, fixed-output physiology, torsades de pointes

## Abstract

Torsades de pointes (TdP) is a polymorphic ventricular tachycardia arising in the setting of repolarization prolongation and is often facilitated by bradycardia-dependent mechanisms. Its management becomes particularly challenging in fixed-output physiology, where rate modulation may conflict with hemodynamic stability. Severe aortic stenosis (AS), especially when accompanied by left ventricular dysfunction, represents such a substrate, requiring a careful balance between electrical stabilization and preservation of forward flow. A 70-year-old woman with symptomatic severe AS and moderate left ventricular dysfunction underwent cardiac computed tomography for preoperative assessment. Immediately following contrast administration, she developed sinus bradycardia, marked QT prolongation (corrected QT, 560 ms), ventricular ectopy characterized by long–short sequences, and recurrent TdP with hemodynamic compromise. Intravenous magnesium and a single bolus dose of amiodarone were administered empirically; however, arrhythmia recurred and QT prolongation persisted. Electrolytes were within normal limits aside from low-normal potassium, which was subsequently corrected. In the setting of bradycardia-dependent TdP and a relative contraindication to aggressive β-blockade in a fixed-output state, semipermanent atrial pacing at 90 bpm was instituted. Rate acceleration suppressed ventricular ectopy and prevented further TdP episodes. Coronary angiography demonstrated no obstructive disease. The patient subsequently underwent urgent surgical aortic valve replacement but developed refractory ventricular fibrillation intraoperatively and could not be resuscitated. This case highlights repolarization vulnerability in structural heart disease, wherein bradycardia-dependent QT prolongation, pharmacologic influences, and procedural stress may converge to precipitate malignant ventricular arrhythmias. Recognition of rate-dependent mechanisms and judicious anti-arrhythmic selection are essential when managing electrical instability in structurally compromised hearts.

**CLINICAL IMPACT:**

Torsades de pointes in patients with severe structural heart disease presents a unique therapeutic challenge when fixed-output physiology limits conventional rate and antiarrhythmic strategies. This case highlights an important and potentially underrecognized interaction between severe aortic stenosis, acquired QT prolongation, bradycardia, and procedural stress. The immediate onset after contrast administration suggests a trigger–substrate interaction rather than contrast exposure as an isolated cause. Importantly, recurrent bradycardia-dependent torsades was successfully suppressed by controlled atrial pacing at 90 beats/min, despite the hemodynamic constraints of severe aortic stenosis. The case also underscores the need to distinguish pause-dependent torsades from other forms of polymorphic ventricular tachycardia, particularly because drugs such as amiodarone may worsen repolarization. Recognition of the underlying electrophysiologic mechanism and judicious use of pacing may be critical for stabilizing patients with malignant ventricular arrhythmias in structurally compromised hearts.

## Introduction

Torsades de pointes (TdP) is a polymorphic ventricular tachycardia arising from delayed ventricular repolarization and is frequently facilitated by pause-dependent mechanisms.^1,2^ The arrhythmia reflects increased dispersion of repolarization and susceptibility to early afterdepolarizations, typically triggered by bradycardia or abrupt cycle-length oscillation in the setting of QT prolongation.^1–4^ While commonly associated with congenital or acquired long QT states, TdP is fundamentally a manifestation of repolarization instability rather than a single etiologic diagnosis.^2^

Management generally centers on correction of reversible triggers; electrolyte optimization; withdrawal of QT-prolonging agents; and, in pause-dependent forms, rate acceleration through pharmacologic or pacing strategies.^1,5^ This framework becomes considerably more complex in fixed-output physiology. In severe aortic stenosis (AS), heart rate is a critical determinant of cardiac output, and both bradycardia and excessive tachycardia may precipitate hemodynamic compromise. Thus, employing tachycardia as a therapy may appear paradoxical in this setting.

We describe a case of refractory TdP in severe AS with marked QT prolongation, in which controlled atrial pacing achieved electrical stabilization despite the constraints of fixed-output physiology. This case highlights the intersection of hemodynamic limitation and repolarization instability in structurally compromised hearts.

## Case presentation

A 70-year-old woman with progressive exertional dyspnea (New York Heart Association class II) was diagnosed with severe calcific AS and moderate left ventricular systolic dysfunction on transthoracic echocardiography. She had no prior history of syncope, documented arrhythmia, or sudden cardiac arrest. There was no known family history of sudden cardiac death or inherited cardiac disease.

She underwent contrast-enhanced cardiac computed tomography for the preoperative assessment of the aortic root and coronary anatomy. A total of 80 mL of intravenous iohexol was administered. Immediately following contrast injection, she developed sinus bradycardia with frequent premature ventricular complexes. This rapidly degenerated into polymorphic ventricular tachycardia consistent with TdP, resulting in hemodynamic instability and transient unresponsiveness **(Figures 1A–1C)**. The chronological sequence of clinical events, interventions, and stabilization is summarized in **Figure 2**.

Electrical cardioversion restored sinus rhythm; however, recurrent TdP episodes followed. Intravenous magnesium sulfate was administered, and an empirical intravenous bolus of 150 mg of amiodarone was given during the acute arrhythmic episode. Due to persistent hypotension, intravenous noradrenaline infusion was initiated for hemodynamic support.

**Figure 1:**
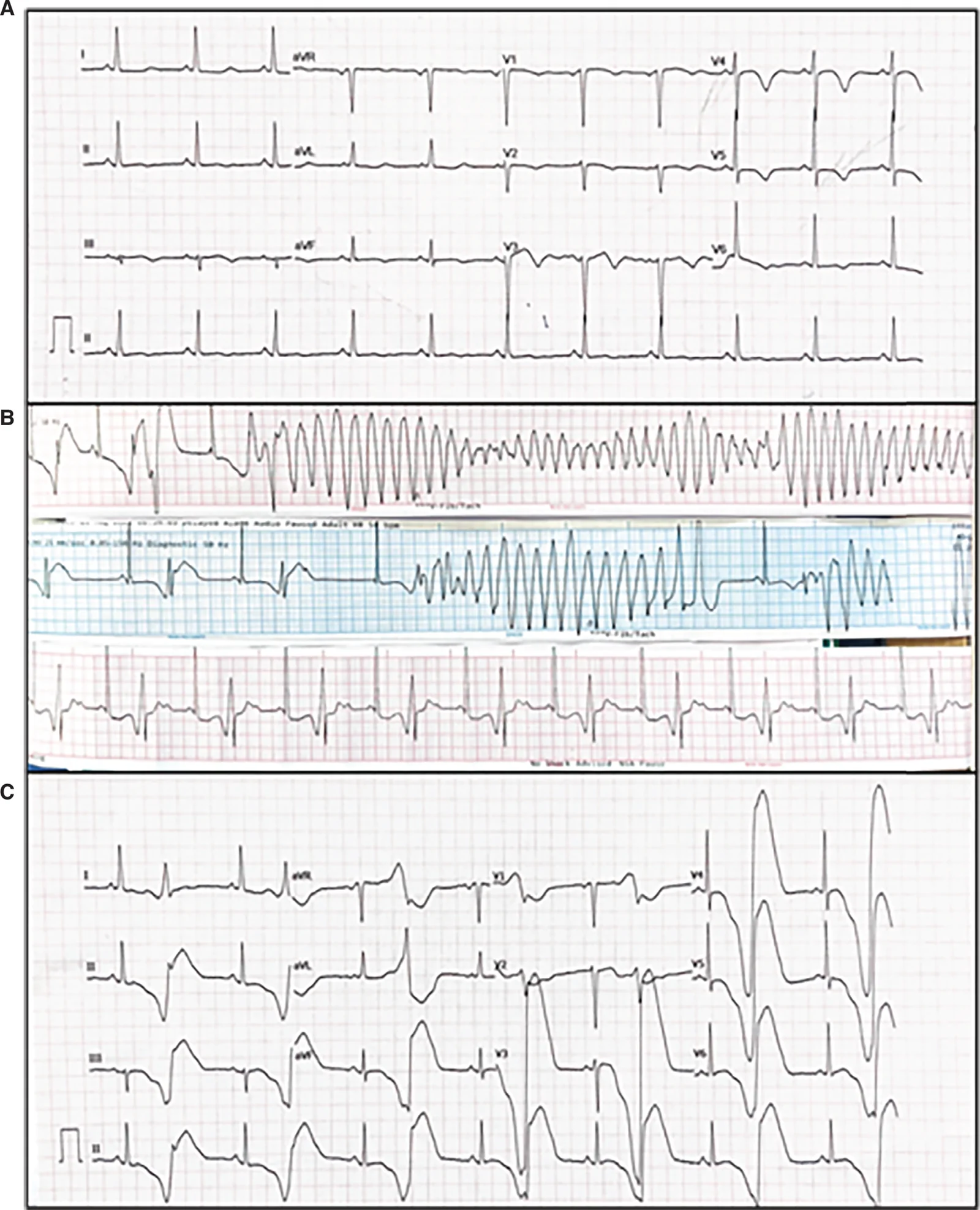
Electrocardiogram of the patient before computed tomography **(A)**, immediately after contrast injection **(B)**, and after cardioversion **(C)**. Tracing analysis **(A–C)**. **A:** Sinus rhythm with significant repolarization abnormality. The QT interval is markedly prolonged (QTc, approximately 560 ms), with broad, delayed T-wave morphology and anterior T-wave inversions in the precordial leads. These findings indicate substantial dispersion of ventricular repolarization. No evidence of ST-segment elevation is present. In the absence of prior tracings, baseline QT status cannot be determined; however, the degree of prolongation establishes a substrate vulnerable to pause-dependent arrhythmia. **B:** Onset of polymorphic ventricular tachycardia following a classic long–short sequence. A premature ventricular complex with a short coupling interval occurs after a compensatory pause, falling on the prolonged terminal portion of the T-wave. This initiates polymorphic ventricular tachycardia with characteristic waxing and waning QRS amplitude and axis rotation consistent with torsades de pointes. **C:** Recurrent episodes with persistent QT prolongation and ongoing ventricular ectopy, further supporting a pause-dependent, repolarization-mediated mechanism rather than ischemia-driven polymorphic ventricular tachycardia. The consistent long–short initiation pattern and subsequent suppression with atrial pacing reinforce the rate-sensitive nature of the arrhythmia.

**Figure 2:**
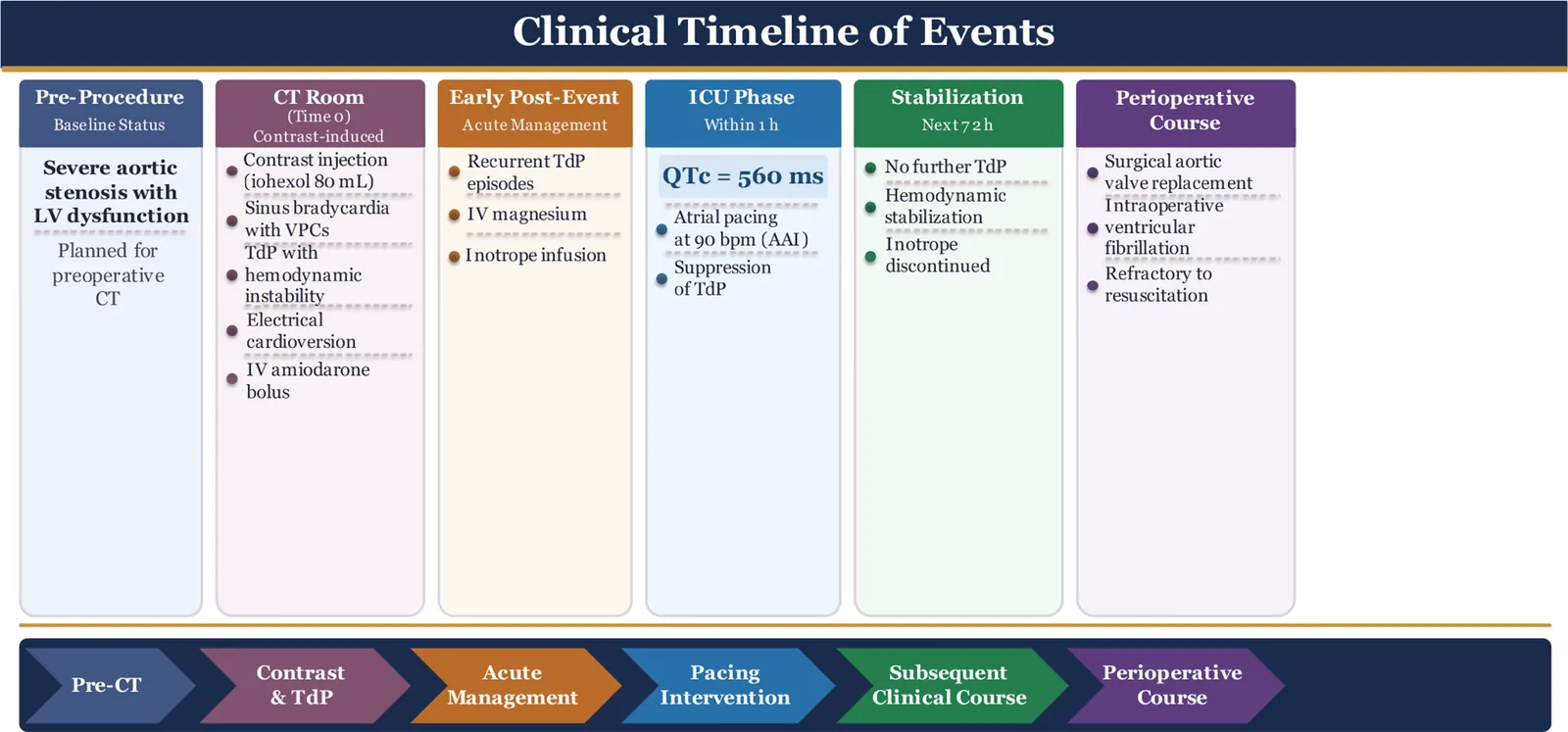
Clinical timeline illustrating the sequence of events from contrast-induced torsades de pointes to pacing-mediated stabilization. *Abbreviations:* CT, computed tomography; IV, intravenous; LV, left ventricular; VPC, ventricular premature complex.

Laboratory testing revealed normal serum calcium and magnesium levels; the potassium level was 3.7 mEq/L and was subsequently corrected and maintained >4.5 mEq/L. Post-event electrocardiography demonstrated marked QT prolongation (corrected QT [QTc], 560 ms). No prior electrocardiograms were available for comparison. Genetic testing for inherited long QT syndrome was not performed.

Despite initial therapy, the patient continued to demonstrate ventricular bigeminy with recurrent TdP in the setting of persistent QT prolongation. Because of recurrent arrhythmia associated with bradycardia, a semipermanent atrial pacing (SPAP) lead was placed via the right internal jugular vein and programmed in AAI mode at 90 bpm. Following the initiation of atrial pacing, the ventricular ectopy resolved, and no further TdP episodes were observed **(Figure 3)**. Hemodynamic status stabilized, allowing the gradual tapering and discontinuation of noradrenaline.

Following stabilization of the ventricular arrhythmia, coronary angiography demonstrated no obstructive coronary artery disease. Given her symptomatic severe calcific aortic stenosis with moderate left ventricular systolic dysfunction, she was subsequently taken for urgent surgical aortic valve replacement (AVR). During the intraoperative period, she developed refractory ventricular fibrillation and could not be resuscitated despite advanced life-support measures.

**Figure 3:**
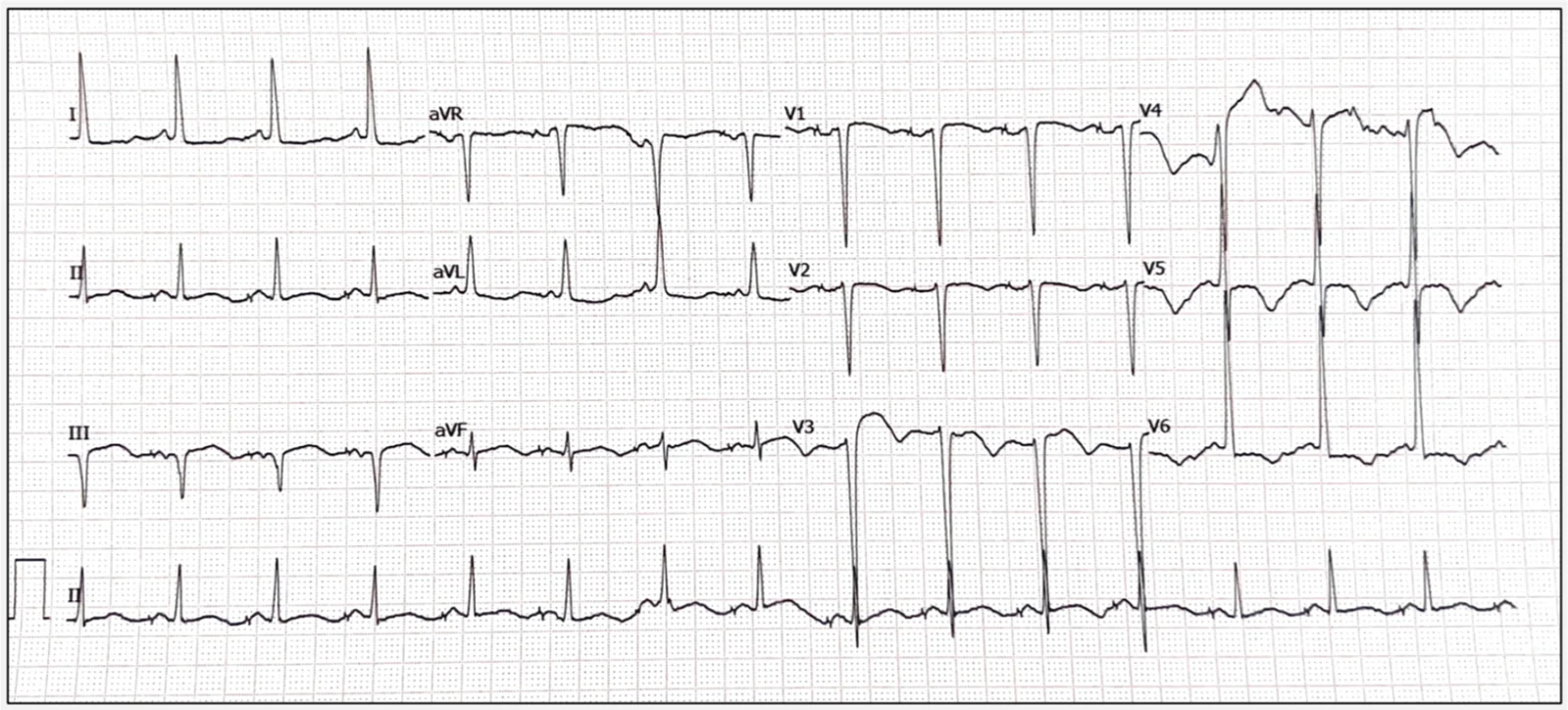
Twelve-lead electrocardiogram of the patient on semipermanent atrial pacing suppressing the torsades de pointes.

Written informed consent for publication was obtained from the patient’s legally authorized representative after her death.

## Discussion

This case illustrates the convergence of structural heart disease, repolarization prolongation, and rate-dependent vulnerability culminating in refractory TdP. Rather than representing a single etiologic diagnosis, the arrhythmia in this patient likely reflected an acquired state of repolarization instability amplified by bradycardia and superimposed procedural stress.

Marked QT prolongation (QTc, 560 ms) was documented following the index event; however, no prior electrocardiograms were available to determine baseline repolarization status. There was no documented family history of sudden cardiac death, and genetic testing was not performed. Accordingly, a diagnosis of congenital long QT syndrome cannot be established. In the absence of such evidence, the more parsimonious interpretation is acquired QT prolongation in the setting of structural heart disease, transient bradycardia, and potential pharmacologic influence.

Severe AS with left ventricular dysfunction provides a substrate that may amplify dispersion of repolarization through hypertrophy, fibrosis, and altered myocardial architecture. In this context, bradycardia assumes particular arrhythmogenic significance. Pause-dependent TdP is well recognized in settings of prolonged repolarization, where long–short cycle sequences facilitate early afterdepolarizations and trigger polymorphic ventricular tachycardia.^1–3^ Prior observations have further demonstrated that the mechanism and context of bradycardia influence susceptibility to pause-dependent TdP^4^ **(Figure 4)**. In our patient, the initial episodes were clearly bradycardia-associated, and suppression of ventricular ectopy following atrial pacing supports a rate-dependent mechanism consistent with these observations.^5^

**Figure 4:**
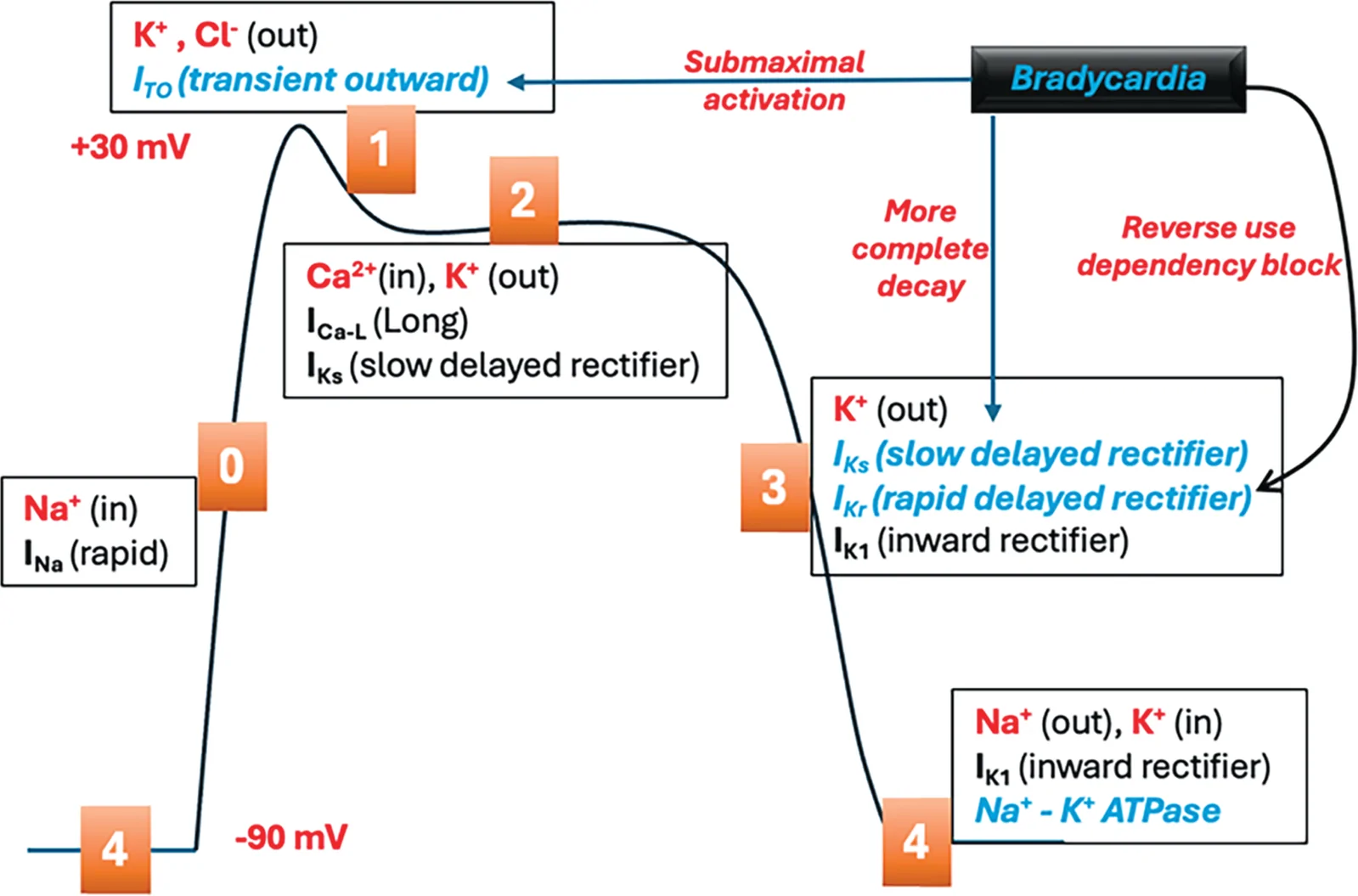
Schematic diagram illustrating the electrophysiological effects of bradycardia on ventricular action potential dynamics.

The temporal relationship between contrast administration and arrhythmia onset raises the possibility of contrast-mediated electrophysiological perturbation. Prior reports describe TdP occurring immediately after intracoronary contrast administration in patients with pre-existing QT prolongation, suggesting that contrast may transiently augment dispersion of repolarization in susceptible myocardium.^6^ However, in most series, the electrophysiological effect is short-lived, supporting a trigger–substrate interaction rather than a sustained primary cause.^6,7^ The persistence and recurrence of TdP in this case therefore suggest that contrast exposure, if contributory, likely unmasked an underlying repolarization vulnerability rather than serving as the sole mechanism.

The administration of intravenous magnesium was appropriate, even in the absence of overt hypomagnesemia, given its stabilizing effect on early afterdepolarizations and its established role as first-line therapy in TdP.^1,2^ In contrast, the empirical use of amiodarone warrants careful reflection. Although frequently employed for polymorphic ventricular tachycardia, amiodarone can further prolong repolarization and may exacerbate pause-dependent TdP.^2^ In retrospect, its administration may have contributed to persistent QT prolongation in this case. This distinction underscores the importance of differentiating between ischemia-mediated polymorphic ventricular tachycardia and bradycardia-dependent TdP when selecting anti-arrhythmic therapy.

Rate acceleration through atrial pacing proved decisive. Increasing the heart rate to 90 bpm suppressed ventricular ectopy and prevented further TdP episodes, consistent with the attenuation of pause-dependent triggers and reduction of cycle-length oscillation.^1,5^ This strategy, however, presents a therapeutic paradox in severe AS. In fixed-output physiology, heart rate is a critical determinant of cardiac output, and excessive tachycardia may compromise diastolic filling and coronary perfusion. Nonetheless, controlled rate acceleration was tolerated in this case and provided effective electrical stabilization.

## Management considerations after semipermanent atrial pacing

Following successful suppression of TdP with SPAP, several strategic pathways were considered **(Table 1)**. One option was early implantation of a dual-chamber implantable cardioverter-defibrillator (ICD) with atrial-based pacing prior to AVR, which would provide QT shortening, reduction in T-wave alternans and dispersion of repolarization, and protection against sudden cardiac death; however, this approach would represent an aggressive invasive therapy before the reassessment of potentially reversible contributors.

**Table 1:** Further Planning After Semipermanent Atrial Pacing with Pros and Cons of Each Strategy

Further Planning After SPAP			
Strategy	Rationale	Pros	Cons
Atrial-based permanent pacing with dual-chamber AICD (DDDR AICD), then send for AVR	QTc shortening T-wave alternans and TDR reduction Permits BB escalation	Proven benefits Protection against SCD (secondary prevention)	Aggressive invasive therapy without drug therapy trial Likely trigger: contrast
Wait for amiodarone washout, then reassess for DDDR AICD before AVR	I_Kr_ is sensitive to amiodarone in a reverse-use–dependent manner: exacerbated the whole scenario	Lessened the TdP risk intra-/postoperatively	Aggressive invasive therapy without drug therapy trial Likely trigger: contrast
AVR and add BB, keep on SPAP, and reassess the need for DDDR AICD after surgery and response to BB	Removal of triggers No contraindication for BB after surgery (low fixed cardiac output resolved by surgery)	Washout of probable drug effect would be achieved Need for AICD can be more objective	Keeping the patient on SPAP indefinitely Unprotected from SCD until AICD implantation Contrast as trigger was presumptive
AVR with LCSD and add BB, keep on SPAP, and reassess the need for DDDR AICD after surgery and response to BB	Same as above Sympathetic trigger removal for torsadogenesis in LQTS	Same as above Added benefit of sympathetic denervation Can be executed with the same thoracotomy settings	Same as above Prolonged and complicated surgery in an already electrically decompensated patient

*Abbreviations:* AICD, automated intracardiac cardioverter-defibrillator; AVR, aortic valve replacement; BB, β-blocker; ECG, electrocardiogram; LCSD, left cardiac sympathetic denervation; LQTS, long QT syndrome; QTc, corrected QT; SCD, sudden cardiac death; SPAP, semipermanent atrial pacing; TdP, torsades de pointes; TDR, transmural dispersion of repolarization.

A second strategy involved awaiting amiodarone washout before reassessing the need for ICD implantation, given the reverse-use–dependent effects of I_Kr_ blockade that may have contributed to persistent repolarization delay. This conservative approach could reduce perioperative arrhythmic risk but would delay definitive protection.

A third approach prioritized surgical correction of the fixed-output state through AVR, followed by β-blocker initiation and continued pacing support, with reassessment of ICD indication after hemodynamic improvement and drug washout. This strategy targeted removal of structural and pharmacologic triggers but required temporary reliance on pacing without immediate defibrillator protection.

Finally, AVR combined with left cardiac sympathetic denervation was considered to further attenuate sympathetic-mediated torsadogenesis, though this would increase surgical complexity in an already electrically unstable patient.

These options reflect the competing priorities of arrhythmic protection, reversibility of triggers, procedural risk, and timing of definitive device therapy.

The intraoperative development of refractory ventricular fibrillation underscores the fragility of the underlying substrate. The precise mechanism cannot be definitively established in the absence of rhythm documentation at onset. The recurrence of repolarization-mediated polymorphic ventricular arrhythmia, including TdP degenerating into ventricular fibrillation, remains a possibility. However, alternative mechanisms such as perioperative hemodynamic instability, transient myocardial ischemia, electrolyte shifts, and anesthetic influences must also be considered. The progression highlights the complex interplay between structural disease, repolarization dynamics, and procedural stress in patients with advanced valvular pathology.

This case therefore emphasizes several clinically relevant points: first, TdP in structural heart disease should prompt consideration of acquired repolarization vulnerability rather than immediate attribution to congenital syndromes; second, bradycardia-dependent mechanisms must be actively sought; third, anti-arrhythmic selection requires careful differentiation between polymorphic ventricular tachycardia subtypes; and, finally, controlled pacing may provide effective stabilization even in the context of fixed-output physiology.

## Conclusion

This case demonstrates the convergence of structural heart disease, acquired repolarization prolongation, bradycardia, and procedural stress culminating in refractory TdP in severe AS. Although the arrhythmia followed iodinated contrast administration, the clinical course supports a trigger–substrate interaction in which the contrast unmasked an underlying repolarization vulnerability rather than acting as a solitary cause.

Controlled atrial pacing at 90 bpm promptly suppressed ventricular ectopy and terminated further TdP episodes, illustrating that rate acceleration can decisively stabilize pause-dependent arrhythmia—even within fixed-output physiology. This therapeutic paradox underscores the importance of mechanism-guided management and judicious antiarrhythmic selection in structurally compromised hearts.

## Data Availability

All data are incorporated into the article.
